# Red wine: A drink to your heart

**DOI:** 10.4103/0975-3583.74259

**Published:** 2010

**Authors:** T.S. Mohamed Saleem, S. Darbar Basha

**Affiliations:** *Department of Pharmacology, Annamacharya College of Pharmacy, New Boyanapalli, Rajampet - 516 126, Andhra Pradesh, India*

**Keywords:** Alcohol, flavonoids, grape juice, polyphenols, resveratrol, wine research

## Abstract

Mortality and morbidity are still high in cardiovascular disease (CVD). Myocardial ischemia reperfusion injury leading to myocardial infarction is one of the most frequent causes of the death in humans. Atherosclerosis and generation of reactive oxygen species through oxidative stress is the major risk factor for CVD. From the literature collection, it has been identified that moderate consumption of red wine helps in preventing CVD through several mechanisms, including increasing the high-density lipoprotein cholesterol plasma levels, decreasing platelet aggregation, by antioxidant effects, and by restoration of endothelial function. The aim of this review is to discuss the accumulating evidence that suggests that red wine possesses a diverse range of biological actions and may be beneficial in the prevention of CVD.

## INTRODUCTION

Since ancient times, cardiovascular disease (CVD) has become a known, life-threatening problem for the world. The risk factors and higher mortality from CVD have been proved without doubt from the well-developed countries of Western Europe, North America and East Asia, as well as for the vast majority of developing countries and even the large urban centers of sub-Saharan Africa.[1] The highest majority of risk factors for this overall mortality are industrial exposure according to their profession, changing dietary habits, lifestyle and increasing obesity. Moreover, tobacco smoking is highly prevalent and risk factors for atherosclerosis tend to occur earlier in life, accounting for earlier presentation of CVD events.[1] CVD is a leading cause of mortality and is responsible for one-third of the global deaths. Nearly 85% of the global mortality and disease burden from CVD is borne by low- and middle-income countries. In India, for example, approximately 53% of the CVD deaths are in people younger than 70 years of age; in China, the corresponding figure is 35%. The majority of the estimated 32 million heart attacks and strokes that occur every year are caused by one or more of the following cardiovascular risk factors – hypertension, diabetes, smoking, high levels of blood lipids and physical inactivity – and most of these CVD events are preventable if meaningful action is taken against these risk factors.[2] The prevalence of coronary artery disease (CAD) in urban North India varies from 7% to 10%[3,4] compared with 3% in USA.[5] The CAD rates in South India are two-folds higher than that in North India, with Kerela reporting 14% in urban and 7% in rural Thiruvananthapuram.[6,7]

A recent report from the World Health Organization (WHO) stated that mortality from CVD in countries of Sothern Asia, including India, Pakistan and Bangladesh, is not as high as that of Central Asian countries, but is significantly higher than that of East Asian countries.[8] Large variation, however, likely exists within these countries between the urban and nonurban populations.

**Figure 1:**
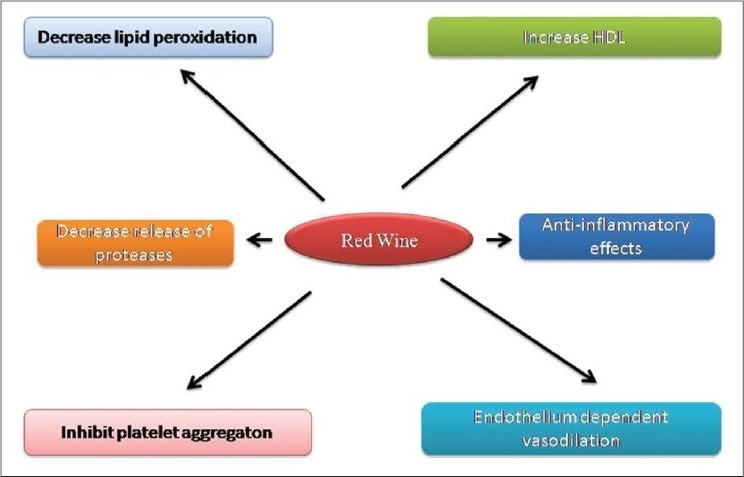
Cardioprotective action of red wine

Epidemiological studies have shown that consumption of foods and beverages rich in natural polyphenols, including those presented in grape fruit, vegetables, tea or red wine, is associated with lower incidence of CVDs and, especially, with ischemic heart disease.[9,10] Moderate wine consumption markedly decreased the cardiovascular and cerebrovascular ischemic events, which has been proven by many epidemiological studies. Red wine may exert its effect by different mechanisms, such as the ability to raise the high-density lipoprotein (HDL) levels, to increase the antioxidant plasmatic potential, to improve endothelium-dependent vasodilation and to inhibit platelet aggregation and leukocyte adhesion [Figure 1]. Particularly, nonalcoholized red wine has a protective mechanism due to its active components like polyphenols, quercetin and resveratrol. The protective mechanism of these components was already proven by many human and animal studies.[11] The present review is aimed at compiling data based on the reported works on red wine and the promising active principles of red wine to prevent CVDs.

## RED WINE:A POTENT ANTIOXIDANT

For many years, the emphasis has been on the relationship between serum total cholesterol levels and the risk of CVD. However, the focus has recently shifted to oxidative stress induced by reactive oxygen species (ROS) and nitrogen-reactive species as important key players in the etiology and pathogenesis of various chronic diseases, including CVD.[12] Antioxidant nutrients are believed to slow down the progression of atherosclerosis due to their ability to inhibit the damaging oxidative processes.[13,14] Epidemiological and prospective studies have shown that consumption of antioxidant vitamins such as vitamin E and ß-carotene could reduce the risk of CVD.[15] Clinical trials also suggest a reduced risk of CVD with vitamin E supplementation.[16] The protective effect of vitamin E can be ascribed to its antioxidant properties. Observations that men and women with CVD show lower levels of circulating antioxidants have led scientists to support the proposed protective role of antioxidants in the prevention and management of CVD.[13] Red wine-active principles like red wine polyphenols, resveratrol and quercetin have experimental cardioprotective properties[17] and may counter one of the mechanisms underlying its antioxidant potential. The cardioprotective properties of individual red wine components are discussed below.

### Red wine polyphenols

Many research and epidemiological studies have shown that intake of polyphenols as grape juice and red wine is associated with a reduced risk of CVD. The most active polyphenol present in red wine is flavonoids, and it is important due to its putative antioxidant properties. The cardiovascular benefits of red wine flavonoids are explained in the French Paradox phenomenon as well as in the Mediterranean diet.[10]

Several studies have documented a protective role of moderate wine consumption (15.5–31 g alcohol/day) in both vascular and nonvascular diseases. Different mechanisms may be responsible for these beneficial effects, including increases in the HDL-cholesterol plasma levels, decreased platelet aggregation, antioxidant effects and restoration of endothelial function by flavonoids. Numerous cross-sectional, observational and controlled studies reveal a range of red wine effects on the different aspects related to CVD. In a recent research, it has been reported that red wine elicits different metabolic, autonomic and endothelial responses among individuals with hypercholesterolemia or arterial hypertension and healthy controls.[18]

Chronic administration of moderate amounts of red wine has been associated with a protective effect on the cardiovascular system.[19] Impaired endothelium-dependent relaxation in both animals and humans is playing a major role in the development of CVD, such as atherosclerosis and hypertension.[20] Generation of ROS is one of the factors for endothelium dysfunction, particularly superoxide anions, which reduce the bioavailability of nitric oxide (NO).[21–24] Although red wine polyphenols have antihypertensive properties, the possibility that they prevent the oxidative stress-induced endothelial dysfunction remains to be determined. In one research, it has been reported that intake of red wine polyphenols prevents Angiotensin (Ang) II-induced hypertension and endothelial dysfunction. Prevention of vascular nicotinamide adenine dinucleotide phosphate (NADPH) oxidase induction and preservation of arterial NO availability during Ang II administration likely contribute to this effect.[19] Red wine polyphenols and a grape skin extract also reduced the blood pressure in N^G^ -nitro-L-arginine methyl ester (L-NAME) and desoxycorticosterone acetate (DOCA) salt-induced hypertensive rats.[25,26]

In another study, it has been reported that chronic administration of resveratrol (a red wine polyphenol) enhances the endothelium-dependent relaxation in spontaneously hypertensive rats, and the protective action might be due to an increase in the bioavailability of NO.[27] In recent research, by using female spontaneous hypertensive rats, it was reported that chronic administration of red wine polyphenols brings about a reduction in blood pressure and vascular dysfunction through reduction in vascular oxidative stress.[28]

Inflammation plays a vital role in the pathogenesis of atherosclerosis, which is a known risk factor for CVD. High levels of fibrinogen and C-reactive protein (CRP), both markers of inflammation, are associated with a risk of developing CVD. In one randomized controlled crossover trial, it has been reported that red wine consumption markedly decreases the level of fibrinogen, but it does not have any effect on the CRP level.[29]

The effects of short-term oral administration of red wine polyphenolic compounds (20 mg/kg/day for 7 days) on the hemodynamics, *ex vivo* cardiac responsiveness and ischemia reperfusion injury were investigated in rats. From this study, it has been concluded that short-term treatment with red wine polyphenols decreases blood pressure and cardiac responsiveness and protects against postischemic infarction via decreased oxidative stress. All the above effects of red wine polyphenols are sensitive to NO synthase inhibition, which implies an involvement of the NO-dependent pathway. This study suggests a basis for the beneficial effects of red wine against CVD.[9] The same research group already reported that the *in vivo* cardiovascular action of red wine and also the oral administration of red wine polyphenols was able to produce a decrease in blood pressure in normotensive rats.[9,29] This hemodynamic effect was associated with an enhanced endothelium-dependent relaxation and induction of the expression of inducible NO synthase and cyclooxygenase 2 within the arterial wall. Moreover, red wine polyphenols accelerated the regression of blood pressure and improved the structural and functional cardiovascular changes, including cardiac fibrosis, in hypertensive rats.[25,30]

In another study, it has been reported that red wine polyphenolic compounds exert a powerful protective effect on the endothelial cells from the injury caused by carbon tetrachloride (CCl_4_). This effect was documented by decreased endothelemia, with corresponded to the diminished endothelial cell swelling and detachment evaluated by histology of the vascular intima.[31] The endothelium-protective effect may be one of the key factors that contribute to the preventive action of red wine on CVDs. Hozumi *et al*.[32] reported that daily intake of red wine polyphenols may benefit patients with or without CVD by increasing the coronary microcirculation. In patients with CAD, 250 ml of de-alcoholized Greek red wine decreased the arterial stiffness and improved the augmentation index, as derived from arterial wave reflection patterns. A similar dose of de-alcoholized red wine decreased the adverse postsmoking arterial wave reflections and lessened the rise in systolic blood pressure. Brachial artery flow-mediated vasodilation was improved by 250–500 ml of de-alcoholized red wine.[33]

Several epidemiological studies suggest that moderate alcohol intake, especially red wine, decreases cardiac mortality due to atherosclerosis. The alcohol effect is described by a J curve, suggesting that moderate drinkers may benefit while abstainers and heavy drinkers are at higher risk.[34] Wine drinkers have higher HDL levels than that of nonwine drinkers. The ingestion of red wine is associated with an increase in the antioxidant activity in the serum, an increase in apolipoprotein A-1 and a decrease in the atherogenic agent lipoprotein (a), mainly due to the presence of flavonoids and stilbenes. It has been further suggested that this increase in the antioxidant activity in patients regularly drinking red wine may be the primary factor inhibiting LDL oxidation, which, in turn, reduces atherosclerotic complications.

## RESVERATROL

Interest in this compound has expanded in recent years, when numerous epidemiological studies showed an inverse correlation between red wine consumption and incidence of CVDs. Accumulating evidence indicates that resveratrol may confer a protective action on the cardiovascular system. The cardiovascular benefits of resveratrol may relate to protecting the heart cells from ischemia reperfusion injury, inhibiting platelet aggregation and decreasing plasma triglycerides and cholesterol accumulation in the aorta. Furthermore, it can also relax the coronary arteries. It seems likely that resveratrol might be partly responsible for the cardiovascular benefits associated with wine consumption.[30,35] Resveratrol is a potent vasodilator and, in several researches, it has been reported that the vasorelaxant properties of resveratrol might be due to NO-mediated relaxation.[36] Novakovic *et al*.[37] reported that resveratrol induces relaxation of the human internal mammary artery (HIMA) rings without endothelium. It seems likely that 4-AP- and margatoxin-sensitive K^+^ channels located in the vascular smooth muscle mediated the relaxation of HIMA produced by resveratrol. In addition, the vasodilator effect of resveratrol through NO-mediated endothelium-dependent relaxation in spontaneous hypertensive rats was also reported.[27] A separate experiment showed that chronic resveratrol administration enhanced the endothelium-dependent vasodilation in ovariectomized, stroke-prone, spontaneously hypertensive rats.[38]

Resveratrol shortened the duration of action potential in papillary muscles in normal guinea pig and also decreased the maximal velocity of phase 0 depolarization in partially depolarized papillary muscles. In addition, resveratrol inhibited delayed-after depolarization and triggered activity induced by ouabain and high Ca^2+^ in the papillary muscle of guinea pigs in a dose-dependent manner.[39–44] In another research, it was found that resveratrol inhibited the spontaneous discharges of neurons in the CA1 area of rat hippocampal slices. These effects were likely due to a decrease of calcium influx.[45] Zheng *et al*.[46] reported that resveratrol decreased the intracellular calcium concentration in rat cardiac myocytes. The inhibition of voltage-dependent Ca^2+^ channel and tyrosine kinase and alleviation of Ca^2+^ release from the sarcoplasmic reticulum (SR) are possibly involved in the effects of resveratrol on rat ventricular myocytes. Intake of resveratrol as red wine also increases the production of platelet-dependent NO and, in this way, it decreases the proinflammatory pathway of p38MAPK thus inhibiting ROS production and, ultimately, platelet function. This activity may contribute to the beneficial effects of moderate wine intake on ischemic CVD.[11]

Resveratrol may exert a protective effect against cell death through many signaling pathways. Hwang *et al*.[47] reported that resveratrol may exert a protective effect on damage to heart muscle through modulation of the AMP-activated kinase (AMPK) signaling pathway. Resveratrol induced a strong activation of AMPK and inhibited the occurrence of cell death caused by treatment with H_2_O_2_. Under the same conditions, inhibition of AMPK using dominant negative AMPK constructs dramatically abolished the effect of resveratrol on cell survival in H_2_O_2_-treated cardiac muscle cells. These results indicate that resveratrol-induced cell survival is mediated by AMPK in H9c2 cells, and may exert a novel therapeutic effect on oxidative stress induced in cardiac disorders.

Ray *et al*.[48] reported that resveratrol can ameliorate myocardial ischemia reperfusion injury. In this research, they found that administration of resveratrol to the rat provides cardioprotection by decreasing the oxidative stress generated in the ischemic-reperfused myocardium. The antiischemic effect of resveratrol in another study states that the resveratrol-treated hearts showed better functional recovery at reperfusion and significant vasodilation, but no variation in high-energy phosphates. This suggests that long-term moderate resveratrol consumption could play an important role in late cardioprotective effects.[49] A preliminary study carried out by the same research group reported that 10 min of resveratrol infusion (10 μM) in Langendorff-perfused rat hearts caused a 40% decrease in the baseline phosphorylation potential without affecting contractility. The level of effluent adenosine was increased by 68%, and paralleled a 50% increase in coronary flow. They suggested that an increase in the adenosine bioavailability is involved in resveratrol-mediated cardioprotection.[49]

The dose-dependent activity of resveratrol was evaluated by Das *et al*.[50] by using the ischemic myocardium in rats. The results thus indicate that at, lower doses, resveratrol exerts survival function by upregulating the antiapoptotic and redox proteins Akt and Bcl-2, while at higher doses, it potentiates a death function by downregulating the redox proteins and upregulating the proapoptotic proteins.

In another study, it has been reported that resveratrol prevents leukocyte recruitment and endothelial barrier disruption induced by a number of superoxide-dependent proinflammatory stimuli, including ischemia and reperfusion, hypoxanthine and xanthine oxidase (HX/XO) or platelet activating factor. These salutary effects appear to be related to the antioxidant properties of resveratrol and contribute to the cardioprotective actions associated with the consumption of red wine.[51]

The protective role of resveratrol in ischemia reperfusion injury is well defined by many researchers. The mitochondrial permeability transition pore (mPTP) opening has been proposed to play an important role in myocardial ischemia/reperfusion injury. The mPTP remains closed during ischemia but opens at the onset of reperfusion, and modulation of the mPTP opening at early reperfusion can protect the heart from reperfusion injury. Because resveratrol protects the heart through a NO-dependent mechanism, and NO has been demonstrated to prevent the mPTP opening, it is possible that resveratrol could modulate the mPTP opening at reperfusion.[52]

## QUERCETIN

Quercetin is one of the most important flavonoids present in red wine. The antioxidant and protective mechanisms in various ischemic conditions were proved by many researches. It has been reported that quercetin inhibited thrombocyte aggregation[53] and had an antihypertensive effect through vasodilator action on the vascular smooth muscles.[54] The studies that focused on the antioxidant efficiency of flavonoids against ischemia/reperfusion (I/R) injury have demonstrated that quercetin possesses robust protective effects in renal, cerebral and hepatic I/R models.[55–57] Quercetin was also demonstrated to improve the contractile function of the left ventricle in experimental myocardial infarction with subsequent 24-h reperfusion.[58] Ikizer *et al*. reported that quercetin has the capacity to protect the myocardial tissue against global ischemia and reperfusion injury. In instances where the molecule is administered for the purpose of acute therapy, this cardioprotective effect of a significant degree can be observed, and the protective action might be due to its antioxidant and cytoprotective actions.

## CONCLUSION

CVDs are now a current major problem in causing mortality in both Western and developing countries. Oxidative stress associated with atherosclerosis and endothelium-dependent vascular inflammation plays a major role in the development of CVD. Red wine contains antioxidative components like resveratrol, proanthocyanidine, quarcetin, etc. and these active components exert protective functions like free radical scavenging effects, decreasing the oxidative stress and reducing the inflammatory atherosclerotic lesion in both animals and humans, which is evident in this review. From these findings, it has been concluded that red wine as a diet supplement might be beneficial for cardiovascular risk factors.
